# Multi-Vortex Regulation for Efficient Fluid and Particle Manipulation in Ultra-Low Aspect Ratio Curved Microchannels

**DOI:** 10.3390/mi12070758

**Published:** 2021-06-27

**Authors:** Shaofei Shen, Xin Wang, Yanbing Niu

**Affiliations:** College of Life Science, Shanxi Agricultural University, Jinzhong 030801, China; wx15581740663@163.com

**Keywords:** curved microchannel, inertial microfluidics, Dean flow, secondary flow, fluid manipulation

## Abstract

Inertial microfluidics enables fluid and particle manipulation for biomedical and clinical applications. Herein, we developed a simple semicircular microchannel with an ultra-low aspect ratio to interrogate the unique formations of the helical vortex and Dean vortex by introducing order micro-obstacles. The purposeful and powerful regulation of dimensional confinement in the microchannel achieved significantly improved fluid mixing effects and fluid and particle manipulation in a high-throughput, highly efficient and easy-to-use way. Together, the results offer insights into the geometry-induced multi-vortex mechanism, which may contribute to simple, passive, continuous operations for biochemical and clinical applications, such as the detection and isolation of circulating tumor cells for cancer diagnostics.

## 1. Introduction

The effective manipulation of fluid and particles is critical to a wide range of applications in biological studies and clinical applications [1,2,3]. As micro-fabrication equipment is improving, microfluidics has generally been known as a progressive technology for fluid and particle/cell manipulation by applying active and passive principles in micro/nano-scale channels [4,5,6]. Inertial microfluidics based on geometry-induced vortexes is recognized as a frequently used passive technology for their prominent merits (e.g., label-and external field-free operating process, high-throughput, increased fluidic controllability, and harmless to cell activity) [7,8,9].

A range of inventive and delicate microchannel geometries that control vortex performance by complying with natural inertial effects at high flow rate have been demonstrated [10,11,12]. The vortex generally generates at (i) spiral or serpentine geometry channel [13,14,15,16,17,18]; (ii) straight channels with expansion–contraction arrays or disturbance obstructions [19,20,21,22,23,24]; and (iii) a multilayer channel with top/bottom arrays [25,26,27,28]. In the mentioned platforms, Dean vortex, Dean-like vortex flows, and Dean-irregular vortex flows can be produced in the main channel section because of the fluid momentum mismatch [12,29]. Furthermore, the horizontal helical vortex can be generated in the straight channels with expansion–contraction arrays under a certain flow rate. Such a vortex has been extensively employed in fluid and particle manipulation (e.g., focusing, mixing, trapping and isolation) [7,9]. However, an effective technique and highly easy production method for achieving a vertical Dean vortex and horizontal helical vortex with uncomplicated configuration have remained largely out of reach.

Accordingly, the present study developed a simple single-layer microchannel by introducing a sequence of order micro-obstacles for simultaneously producing a helical vortex and Dean vortex to achieve the manipulation of fluid and particles with high efficiency [30,31,32]. The unique semicircular channel design with ultra-low aspect ratio (AR = 1:9) mitigated the challenge of production-technology and fabricating-cost increase by employing standard soft lithography and stereolithography technology. To improve the performance and throughput of processing, the use of multi-vortex formation in curved microfluidic horizontal and vertical planes was primarily highlighted. The unique mechanism of multi-vortex and underlying physics in the semicircular channel was studied systematically. The prominent fluid mixing and dynamic concentration gradient could be achieved by regulating operational modes. Efficient media exchange could be achieved in the designed microchannels, which exhibited less sensitivity to the high flow condition.

## 2. Materials and Methods

### 2.1. Materials and Reagents

RTV 615 poly(dimethylsiloxane) (PDMS) pre-polymer and curing agent were purchased from Momentive Performance Materials (Waterford, NY, USA); surface-oxidized silicon wafers were from Shanghai Xiangjing Electronic Technology, Ltd. (Shanghai, China); SU8 photoresist and developer were from Microchem (Newton, MA, USA); the analytical reagent-grade solvents and other chemicals were purchased from local commercial suppliers, unless stated otherwise. All solutions were prepared using ultra-purified water supplied by a Milli-Q system (Millipore®, Burlington, MA, USA).

### 2.2. Device Design and Fabrication

The microfluidic devices utilized for this study were fabricated using standard soft lithography with PDMS. First, patterns for the microchannels were designed using AutoCAD software. Second, microchannels were printed on transparent films (MicroCAD Photomask, Ltd., Suzhou, China) to form a photomask. As a result, the mold was fabricated through a single step under UV light using an SU8 Photoresist on a BG401A mask aligner (7 mW cm^−2^, CETC, Beijing, China). Before fabricating the microfluidic device, the mold was exposed to trimethylchlorosilane vapor for 3 min. A well-mixed PDMS pre-polymer (RTV 615 A and B (10:1, *w*/*w*)) was poured onto the mold placed in a Petri dish to yield a 3 mm-thick PDMS replica. After degassing, the mold was baked at 80 °C for 50 min. The PDMS replica was then peeled off the mold. Holes for inlets and outlets were punched with a metal pin. Afterward, the PDMS replica was trimmed, cleaned, and placed on a clean glass slide (3000 rpm, 60 s, ramp 15 s) with a PDMS pre-polymer (RTV 615 A and B (20:1, *w*/*w*)) cured for 20 min in an oven at 80 °C. Finally, the microfluidic device was ready for use after baking at 80 °C for 48 h.

### 2.3. Numerical Simulation

In order to assess fluid motion in the microfluidic control system, ESI-CFD software was used to simulate the computational fluid dynamics (CFD) (V2016.0, ESI CFD, Inc., Huntsville, AL, USA). The steady-state incompressible flows were applied as described in the section “Theory and Design Principle” to explain the formations of helical vortex and Dean vortex. The transient-state incompressible flows are verified in the section “Fluid Mixing Characterization and Applications”. In this experiment, various flow rates were set at the input end, and the outlet was set as a fixed pressure boundary condition. There was no slip boundary condition on the groove wall. For the exploration of fluid phenomena in the microchannels, FLOW and CHEM modules in CFD-ACE+ were employed. Multiblock structured meshes of around two million cells were employed. On account of the finite volume means, the conservation of Navier–Stokes momentum in our system is defined by Equation (1) as follows:

(1) $$\frac{\partial }{\partial t}\left(\rho \vec{V}\right)+\nabla \cdot \left(\rho \vec{V}\vec{V}\right)=-\nabla P+\nabla \cdot \overset{=}{\tau }$$

The conservation of mass is described by the continuity Equation (2) as follows:

(2) $$\frac{\partial \rho }{\partial t}+\nabla \cdot \left(\rho \vec{V}\right)=0$$

where *ρ* is fluid density; $\vec{V}$ is velocity vector; *P* is pressure; $\overset{=}{\tau }$ is stress tensor; *t* is time; and ∇ is the standard spatial grad operator. The physical properties of water were applied to the fluids participating in the simulation (density *p* = 1000 kg m^−3^ and dynamic viscosity *µ* = 10^−3^ kg m^−1^ s^−1^). A diffusion coefficient D = 10^−10^ m^2^ s^−1^ was used for the fluids in the simulations. The convergence limit and iteration were set to 10^−4^ and 10^4^ time steps until the flow reached the outlet. In addition, for fluid mixing calculation, waters A and B were set as 0 and 1, respectively. A second order limiting scheme was used for solving the species’ diffusion. The convergence limit for mass fraction was set to 10^−6^ and the simulations were run for ~2000 time steps until flow reached the outlet. 

### 2.4. Sample Preparation

For microfluidic particle manipulation, a solution containing fluorescent polystyrene particles (Phosphorex, Inc. Shanghai, China) was used. The particles were 30 μm (σ_p_ = 3.19 μm) in diameter and were labeled with FITC fluorophores. The suspension of the fluorescent particles was prepared by diluting the particles in ultra-purified water (supplied by a Milli-Q system, Millipore^®^) containing 0.5% *w*/*v* Tween 20. Prior to each experiment, the particle suspension in a 15 mL vial was sonicated for at least 8 min to achieve a sufficiently mono-dispersed suspension. The particle stream position was determined by analyzing the grayscale line scanned across the channel width.

### 2.5. Fluid and Particle Application Experiments

To monitor the mixing dynamics in the designed channel, fluorescein (100 µM fluorescein in NaHCO3 buffer, pH 8.3) was used in the present study as a model cue to visually characterize the molecular distribution. In addition, with the use of a syringe pump, this study conducted the introduction of the fluorescein solution and fresh NaHCO3 buffer to the channel. Each experiment was repeated at least ten times. Then, in order to quantify normalized intensity under different flow conditions, the images from the inlet and outlet were converted to grayscale images, and then analyzed by drawing a perpendicular line across the channel to obtain their intensity values.

The variation coefficient of CoV = *σ_c_*/*μ* refers to the rate of standard normalized concentration (*σ_c_*) deviation to the mean concentration (*μ*). *σ_c_* and *μ* were calculated by

(3) $$\sigma_{c}=\sqrt{\frac{\left[\sum_{i}^{n}{\left(C_{i}-\mu \right)}^{2}\right]}{n}}$$

(4) $$\mu =\frac{1}{n}\overset{n}{\underset{i}{\sum }}C_{i}$$

In the equation, *C_i_* represents the concentration in the respective datum point (*i*); *n* expresses the overall data point number. CoV represents a measure for the species’ dispersion in the relevant region. Specific to no mixing, CoV = 1, and specific to complete mixing, CoV = 0. A comparison was drawn between the experiment-related CoV data and the results achieved through the simulation.

In terms of the particle application experiments, a mixture of solutions covering fluorescent polystyrene particles (10^5^ particles per mL) and fluorescein solutions (100 µM fluorescein inside NaHCO_3_ buffer, with the pH value of 8.3) received the introduction to the channel from inlet 2. Moreover, under the identical flow ratio, from inlet 1, the fresh NaHCO_3_ buffer received the simultaneous injection. The separation efficiency of fluorescent particles in fluorescein solution and the purity of fluorescein collected from the target outlet was calculated by

(5) $$\mathrm{Separation}\;\mathrm{efficiency}=\frac{n_{outlet\;1}\;}{n_{inlet\;2}}\times 100$$

(6) $$\mathrm{Purity}=\frac{N_{outlet\;1}}{N_{inlet\;2}}\times 100$$

In the equation, *n_outlet_*_1_ denotes the number density belonging to target particles from the target outlet 1; *n_inlet_*_2_ represents target particles’ number density within the original sample; *N_inlet_*_2_ expresses the fluorescence intensity of fluorescein solutions collected within the original sample; *N_outlet_*_1_ is the fluorescence intensity pertaining to fluorescein solutions collected from the target outlet 1. 

### 2.6. Experimental Setting and Approach

During each experiment, the sample was pumped into the microfluidic device at a flow rate using a syringe pump to generate a stable and continuous microflow; either a 1 or 10 mL syringe was connected to the device using Tygon tubing (internal diameter: 0.42 mm; length: 25 cm). Prior to use, the device system was initially irradiated with UV light for 1 h, and then sequentially rinsed with 70% ethanol, followed by ultra-purified water and a PBS working buffer. An inverted microscope (Olympus, CKX41, Tokyo, Japan) with a charge coupled device camera (Olympus, DP73, Tokyo, Japan) and a mercury lamp (Olympus, U-RFLT50, Beijing, China) was used to obtain phase contrast and fluorescence images. Image and data were processed and analyzed using Image-Pro^®^ Plus 6.0 (Media Cyternetics, Silver Spring, MD, USA), origin 9 (origin Inc. Shanghai, China) and SPSS 12.0 (SPSS Inc. Shanghai, China). The results and error bars in the graphs were expressed as the mean ± SD. Tests of data significance were performed using one-way analysis of variance (ANOVA).

## 3. Results and Discussion

### 3.1. Theory and Design Principle

The helical vortex, generally known as a unique rotational flow arising in the straight channels with expansion–contraction arrays or disturbance obstructions, is induced by the obstructions within the channel horizontal plane [7,8,33]. The helical vortex is a rotational flow that exists in the horizontal plane of the channel [34,35]. The existence of a helical vortex is distinct from vortex flow in the separation zone of the straight channel and dean vortex in the curved channel [36,37,38]. The formation of helical vortexes is determined by a number of factors (e.g., the ratio of the contraction area to the expansion area, shape and size of the obstruction, and the flow velocity, as well as the fluid inertia) [8,9,11]. Notably, the fluid inertia is largely dependent of the Reynolds number (*Re*). The Dean vortex induced in the vertical planes of the curving channel by a pressure gradient refers to a notable inertial effect. It is attributed to the inconsistency of fluid momentum in the center of the channel and the area close to the wall. The magnitude and qualitative aspects of the Dean vortex are characterized by the dimensionless parameter Dean number *De* = *Re* (*D_h_*/2*R*)^0.5^. The strength of the Dean vortex is dependent on the geometrical ratio *D_h_*/2R and the magnitude of the underlying downstream flow (*Re= ρUD_h_/μ*), where *Re* denotes the channel Reynolds number, *R* expresses the curvature radius, *D_h_* represents the hydraulic diameter of the channel, *ρ* denotes the fluid density, *U* expresses the maximum velocity in the channel and *μ* represents the fluid viscosity [8].

As indicated from the mentioned analysis, the qualitative characteristics and magnitude of two vortexes are significantly impacted by channel construction apart from *Re*, so they act as the vital factors to design the channel structure for preferable fluid and particle applications. To be more specific, the parameters of microchannel construction (e.g., the ratio of *D_h_* to *R*, AR (H/W, H represents the channel’s height and W represents the channel’s width), as well as the various H of the inner and outer wall) significantly impact the distribution and strength of multi-vortex [7,8,9]. However, the existing strategy of designing geometry for producing a multi-vortex necessitates a high-AR expansion–contraction channel construction, because of the strengthening of the Dean and helical vortex effect with the increase in height [30,31]. To more effectively explore the inertia mechanism of the multi-vortex within a low AR microchannel, a semicircle microchannel was developed with an ultra-low AR (H/W = 100 μm/900 μm) and 6000 μm radius of curvature. Furthermore, the arranged micro-obstacles in the channel (i.e., dimension-confined curved channel, D-channel) acted as a dependent regulator to regulate the distribution and magnitude of the multi-vortex. The arranged micro-obstacles were only introduced to the inner wall of the channel, so the Dean vortex generated in the normal semicircle channel could be more significantly exploited for fluid and particle manipulations [30,31]. Four D-channels (D1, D2, D3 and D4) with the identical channel length were developed (Figure S1), exhibiting the same size and the different amounts of ordered micro-obstacles [2,6,14,30]. 

**Figure 1:**
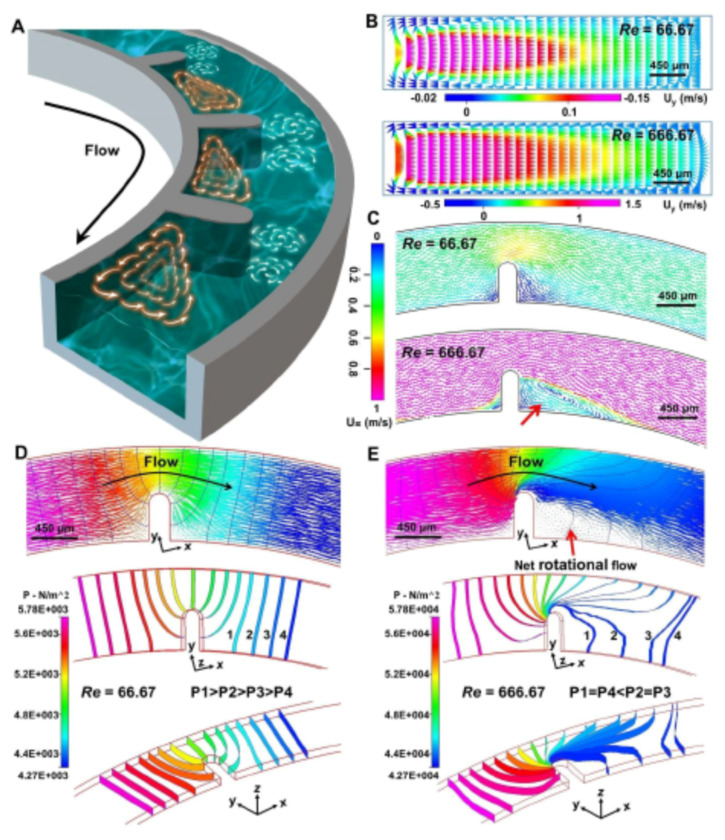
Characteristic of multi-vortex formation based on dimensional confinement: (**A**) schematic diagram of multi-vortex formation based on dimensional confinement; (**B**,**C**) simulated characterization of Dean vortex (**B**) and helical vortex (**C**) using computational simulation method of the FLOW module under different flow conditions. The red arrows represent vortex distributions. The U_y_ represents the fluid velocity field in vertical plane points to the channel wall. The U_x_ represents the fluid velocity field in the horizontal plane refers to the main flow direction from the inlet to the outlet; (**D**,**E**) pressure distributions under low (**D**) and high (**E**) flow conditions around a micro-obstacle under multi-angle view. The arrow colors represent the value of fluid pressure.

Our group has systematically investigated the regulation mechanism of the Dean vortex and helical vortex in the ultra-low AR-curved microchannels [30,31,32]. The Dean vortex could be elevated through the decline of channel height in semicircular channels. In addition, the prominent acceleration of the Dean vortex has been demonstrated by up-regulating the operational flow rate. Consistent with previous observations, this phenomenon is especially obvious since the high flow condition (*Re* = 666.67) can result in a significant acceleration of the Dean vortex (Figure 1B). Numerical simulation regarding pressure distributions under various flow conditions was further employed to qualitatively study the unique formation mechanism of the helical vortex in the curved channel (Figure 1D,E). Under low flow conditions (*Re* = 66.67), as demonstrated by the shape and pressure value of isobaric surfaces around the micro-obstacle, pressure distribution was symmetric and organized (P1 > P2 > P3 > P4). Under a high flow condition (*Re* = 666.67), however, the mentioned isobaric surfaces were significantly deformed as impacted by asymmetric pressure distribution. With the presence of inertia, a complex low-pressure distribution was generated (P1 = P4 < P2 = P3) behind the micro-obstacle. For the aforementioned reason, when fluid from high-pressure regions moved towards these regions, the net rotational flow, i.e., helical vortex (red arrow) was produced to conserve the mass balance. Furthermore, obvious helical vortex formation in the horizontal plane of the designed channel was identified (Figure 1C). These findings proved the significance of using appropriate operational flow conditions in sequenced micro-obstacles of curved channels for the modification of multi-vortex achievement. In subsequent sections, in-depth studies on the responses of the multi-vortex to regulation for fluid and particle manipulation based on the devices are presented.

### 3.2. Fluid Mixing Characterization and Applications

**Figure 2:**
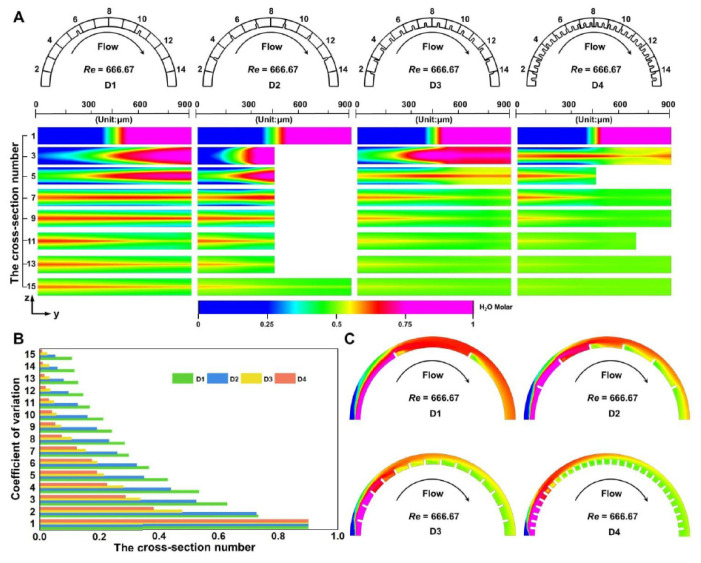
Simulated characterization of multi-vortex regulation for fluid mixing in four dimension-confined curved microfluidic channels (D1, D2, D3, D4) using computational simulation method of volume-of-fluid module. The three-dimensional fluid mixing solution was obtained using the full three-dimensional incompressible Navier–Stokes equations, and the calculated flow conditions of 10 mL/min (*Re* = 666.67) were then used to solve the convection–diffusion equation for species transport. Red and blue represent the normalized concentrations of 1 and 0, respectively: (**A**) simulated comparison of concentration distribution in 15 cross-sections of the four D-channels. The four dimension-confined curved microfluidic channels are divided into 15 cross-sections at equal interval, respectively; (**B**) quantitative analysis of fluid mixing by CoV values in 15 cross-sections of the four D-channels. The results correspond to the cross-sections in Figure 2A; and (**C**) simulated comparison of the concentration distribution in the x–y plane (located at a height of 50 μm in the four D-channels) under same flow conditions.

Fluid mixing is recognized as a vital operation during chemical and biochemical processes. However, it is insufficiently accurate to achieve in the low aspect ratio microchannels under high operational flow conditions. This is because of the reduction in Dean vortex effect as the height decreases and the adverse effect exerted by reacting time reduction with the rise of the flow rate. To overcome those mixing challenges, multi-vortex regulation generated by dimensional confinement, such as helical vortex in the horizontal plane and Dean vortex in the vertical plane induced by micro-obstacles in the curved channel, can improve fluid mixing. To explore how multi-vortex regulation influences fluid mixing under high operational flow conditions, a comparative experiment of fluid mixing by employing a volume-of-fluid module was performed in the four D-channels (Figure 2A). The computational results demonstrated that the mixing effect appeared improved, accompanied by the increase in micro-obstacles, especially for the consequence in D3 and D4 which are superior to that in D1 and D2. As previously demonstrated, since the D4 has the lowest magnitude of helical vortex and Dean vortex [32], the maximum number of multi-vortex productions generated by dimensional confinement indeed advanced the effect of fluid mixing. Similar results can be more noticeably found by the quantitative comparison of coefficient of variation (CoV) values in four D-channels (Figure 2B). The smaller the resulting CoV values, the better the mixing effect would be. To be specific, whenever the fluid passed micro-obstacles of four D-channels, the mixing effects could be significantly strengthened (Figure 2C and Movie S1, ESI†). This result is explained as generating a multi-vortex by micro-obstacles could expedite the mixing and allow the fluid to realize molecular diffusion under a shorter time and distance. In brief, the strategy of designing ultra-low aspect ratio curved microchannels based on dimensional confinement for producing multi-vortex regulation is considered highly feasible in order to bring a powerful and energetic fluid mixing effect in a high-throughput, highly efficient, and easy-to-use manner. This semicircular type of microprocessor does not require higher pressures to handle without bringing in more expensive pumps and more rugged chips, meaning that it is has promising to establish various portable and low-cost lab-on-a-chip platforms.

**Figure 3:**
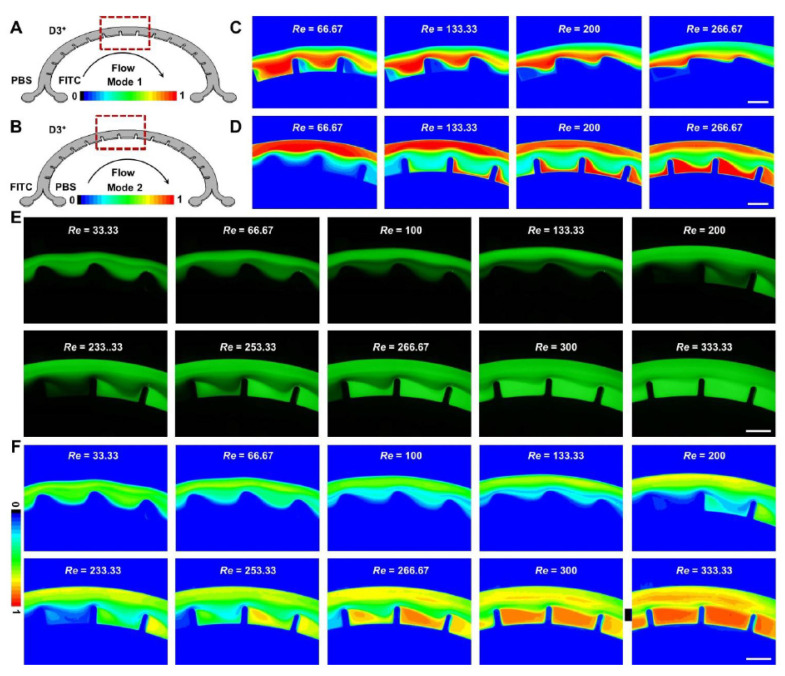
Fluid manipulation characterization of multi-vortex regulation in the two dimension-confined curved microfluidic channels (D3+ and D3++): (**A**,**B**) the operation mode 1 (**A**) and mode 2 (**B**) in the D3+ channel. Red dotted lines are used to analyze fluorescein distributions at the same positions. The analytical results are listed in Figure 3C,D; (**C**,**D**) contour diagram formation for the fluorescein distributions in mode 1 (**C**) and mode 2 (**D**) under certain flow conditions; (**E**) experimental results of fluorescein distributions in mode 3 under different flow conditions; and (**F**) contour diagram formation for the fluorescein distributions in mode 3 under different flow conditions. Scale bars, 600 μm.

**Figure 4:**
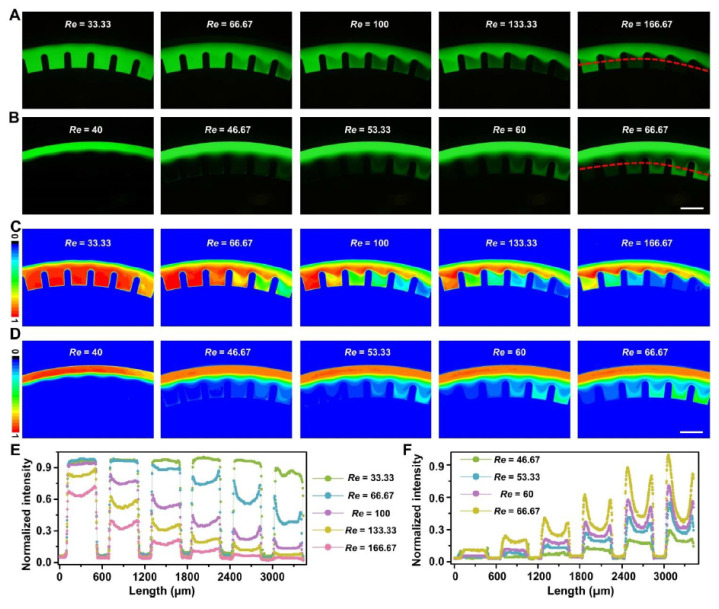
Fluid manipulation characterization of multi-vortex regulation in the two operation modes (mode 4 and mode 5) of the D4+ channel: (**A**,**B**) experimental results of fluorescein distributions in mode 4 (**A**) and mode 5 (**B**) under different flow conditions. Red dotted lines are used to analyze fluorescein distributions at the same positions. The analytical results are listed in Figure 4E,F; (**C**,**D**) contour diagram formation of fluorescein distributions in mode 4 (**C**) and mode 5 (**D**) under different flow conditions; (**E**,**F**) quantitative evaluation of fluorescein distributions in mode 4 (**E**) and mode 5 (**F**) under different flow conditions. Scale bars, 600 μm.

Furthermore, the dynamic generations of concentration gradient were observed in the chambers (the space between two micro-obstacles) of optimized D-channels (Figure 3A,B and Figure S2, ESI†). The contour diagrams (Figure S3, ESI†) were adopted to describe the accurate concentration-gradient distributions of fluorescein. As indicated from the analytical and statistical results from thousands of imaged fluorescein trajectories, various concentration gradients could be formed under different operation modes of 1–5 (Figure 3C–F and Figure 4). Specifically, the interesting phenomena in operation mode 3 (Movie S2, ESI†), mode 4 (Movie S3, ESI†), and mode 5 (Movie S4, ESI†) were significantly conspicuous. A series of concentration gradients in the chambers were stably constructed in operation mode 3 (Figure 3E,F) under a wide range of flow conditions (*Re* = 200–333.33), operation mode 4 (Figure 4A,C) under a wide range of flow conditions (*Re* = 33.33–166.67) and operation mode 5 (Figure 4B,D) under a wide range of flow conditions (*Re* = 46.67–66.67). It is noteworthy that under the altered flow conditions, fluorescein had various concentration distributions. The smaller operational *Re* might produce stronger fluorescein concentration in the chambers by mode 4 (Figure 4E), whereas the resulting fluorescein concentrations in the same chambers by operational mode 5 were reverse (Figure 4F). Thus, by regulating the operation modes, easy-to-operate concentration-gradient constructions in the identical batch of chambers could be achieved from small to large or the opposite. The results showed that the adjustment of the multi-vortex in ultra-low AR curved channels by local confinement has great potential in the controllable mixing and concentration-gradient constructions of fluid manipulation with high throughput (mL/min), which is technologically and functionally comparative to the previous strategy that achieves fluid applications with low throughput (μL/min) [7,8,9,34]. Further study of the particle manipulation characterization and applications based on the devices are presented in subsequent sections. 

### 3.3. Particle Manipulation Characterization and Applications

**Figure 5:**
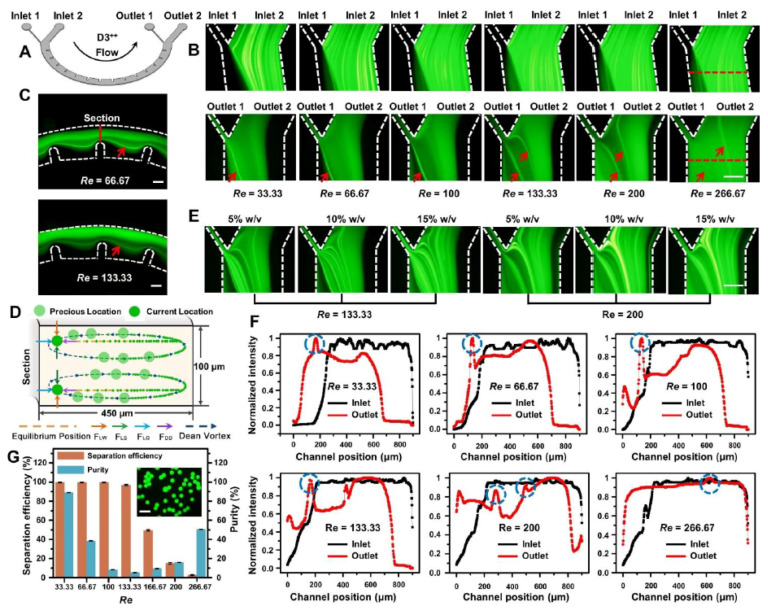
Particle manipulation characterization and exchange of the carrier medium by multi-vortex regulation in the D3++ channel. A mixture of solutions containing fluorescent particles and fluorescein solutions was introduced from inlet 2 into the channel, and the fresh NaHCO3 buffer was simultaneously injected from inlet 1 at the same flow rate using a syringe pump: (**A**) configuration of the D3++ microfluidic device for the specific exchange of the carrier medium. The detailed design parameters can be found in Figure S1; (**B**,**C**) trajectories of fluorescent particles and fluorescein in the inlet and outlet regions (**B**) and middle regions (**C**) of designed channel under different flow conditions. Scale bars, 300 μm; (**D**) schematic illustration of particle and fluorescein separation in the proposed D3++ channel. The way of particle and fluorescein migration is determined by balancing the magnitudes of four forces: (1) shear-induced lift force (FLS), (2) wall-induced lift force (FLW), (3) Dean drag force (FDD), and (4) Saffman lift force (FLΩ). Inertial particle focusing arises from inertial lift (FL), Dean drag forces (FDD), and Saffman lift force (FLΩ). It has been commonly understood that the FLs are composed of the FLS and FLW. FLΩ is a hydrodynamic force initiated from the particle rotation, which is very weak and nearly fails to work in the ultra-low AR channel. Therefore, the two dominant competitions of FL and FDD promote the particle to move with the Dean vortex along the equilibrium lines until drawing near inner channel wall; (**E**) trajectories of different *w*/*v* (5–15%) particles under different flow conditions in the outlet region; (**F**) fluorescence intensity analysis of the particle and fluorescein trajectories under different flow conditions in the outlet region. The results correspond to the red dotted lines in Figure 5B. The blue dotted circles represent the position of particles. The fluorescence intensities of the distributed particle and fluorescein indirectly represent particle and fluorescein locations, which can be precisely analyzed in terms of their distribution characteristics; (**G**) Separation efficiency of fluorescent particles and purity of fluorescein collected from the target outlet under different flow conditions. Standard deviations deduced from ten parallel experiments are shown as the error bars. Inset shows the stained particles which were collected from the outlet 1 at a high flow rate of 2 mL/min (*Re* = 133.33). Scale bars, 40 μm.

In the following, we took advantage of multi-vortex regulation in the optimal dimension-confined D3 channel (Figure 5A) to perform particle-relevant manipulation characterization and applications. We introduced the fluorescein-containing fluorescent particle solution and the sheath flow of the NaHCO_3_ buffer from two inlets into the channels under different flow conditions (Figure 5B). The steady particle localization could be gradually developed and finely collected in outlet 1 under a wide range of high flow rates (0.5–2 mL/min, *Re* = 33.33–133.33). No clogging problem happened in our device under either low (Movie S5, ESI†) or high (Movie S6, ESI†) flow conditions due to the large-scale dimension of microchannels (W ≥ 450 μm). Interestingly, as the wide dimensions and narrow dimensions were successively conserved across the channel, the particles and fluorescein solution alternately presented unique focusing promotion and focusing equilibrium under the flow conditions of high speed (Figure 5C). When the fluorescein-containing fluorescent particle solution flowed along the inner wall of the channel by a sheath flow, various hydrodynamic forces acted on the particles (Figure 5D). The small fluorescent molecules solely underwent Dean migration within the channel, which was dominantly influenced by the Dean vortex rather than the inertial lift forces (FL). Consequently, the inertial lift forces drove fluorescent particles toward the inner wall and the Dean drag forces (FDD) entrained the fluorescent solution to outer wall (Figure 5D,F). Thereby, the fluorescent particles and fluorescent solution were migrated in opposite directions along the channel and finally isolated via the bifurcation of the outlet region reaching outlet 1 and outlet 2, respectively; thus, the carrier medium was finally exchanged. The results demonstrated that the Dean vortex regulation induced by the micro-obstacles could significantly encourage FDD, thereby accelerating particles and fluorescein solution by only focusing passing a semicircular channel, as compared to our previous inertial studies [30,32]. 

Furthermore, another phenomenon in inertial microfluidics should be considered when designing a device at a high *Re*. As the particles and fluorescein solution escaped from the narrow regions to expansion regions, they might also be influenced by the helical vortex in the horizontal plane of expansion regions at high operational flow rates. The induced helical vortex area might play an important role as a void area in the expansion regions, which could generate additional FL by applying the particles and speed up to the particle focusing and fluid-mixing process. For example, at higher flow rates (over 2 mL/min, *Re* ≥ 200), particle stream was further pushed away from the inner wall and outlet 1. Fluorescein solution was split into double streams at *Re* = 200. With the flow rate increasing to 3 mL/min (*Re* =266.67), fluorescein solution could not be formed focusing and thoroughly mixed with the sheath flow (Figure 5D,F). This is because the induced high-speed multi-vortex could significantly promote the mixing progress via a smaller mixing path. The helical vortex as a dead volume could deform the curvature of the curved path at the entrances of the contraction and expansion regions, thereby breaking the symmetry in Dean flow and decreasing the transverse velocity, which adversely affected the formation of the Dean vortex [24,39]. The mixed fluorescein-containing fluorescent particle solution presented a wide range of trajectories for passing in the designed device with the function of multi-vortex regulation and hydrodynamic forces, providing potential approaches for sample focusing and sorting.

Then, the higher particle concentration effects (5–15% *w*/*v*) on the exchange of the carrier medium in the outlet regions were observed under the identical operation conditions (Figure 5E). It was found that particles cannot all be focused and migrated to the target outlet 1, primarily because the ascending numbers of particles may exaggerate particle interactions and cause the deterioration of focusing imbalance. Additionally, the migration effect to the outlet 1 at *Re* = 133.33 was significantly higher than that at *Re* = 200, suggesting that the device can present a better focusing performance by setting optimal operational flow conditions. Finally, to evaluate the effectiveness of carrier medium exchange, the separation efficiency of fluorescent particles and the purity of fluorescein collected from outlet 1 were calculated under different flow conditions. As can be seen from Figure 5G, the lowest and highest separation efficiencies of fluorescent particles were as high as 96.92% and 99.65% when the operational conditions ranged from *Re* = 33.33 to 133.33, respectively. The lowest and highest purity of fluorescein were 5.41% and 9.60% when the operational conditions ranged from *Re* = 100 to 166.67, respectively. Compared with prior exchange techniques of carrier medium [6,7,8,9], where media exchange only occurred at specific low flow rates, the present system achieving media exchange was less sensitive to the flow condition, in particular high flow rates (mL/min). This characteristic demonstrates a benefit with chemical engineering and biochemical applications such as the rapid exchange and handling of reagents, which is defining a range of flow conditions to choose for manipulation, instead of setting a fixed flow rate to yield optimal manipulation. Although we cannot acquire pure particle extraction where the fluorescent solution was completely eliminated, the simple channel structure allows for easy configuration by the multiplex cascading of designed circuits in series, with the ability to analyze milliliter-scale samples in a high-throughput and highly efficient manner. Furthermore, the unique layout of two inlets (sample–inlet width: sheath–inlet width = 9:1) under the same operational flow conditions is beneficial for reducing the waste of sheath flow required and convenient to operate with a syringe pump, which is superior to the achievements based on the current media exchange technologies [7,8,9]. 

## 4. Conclusions

In this study, we investigated the geometry-induced generation and regulation mechanism of a multi-vortex under different operational flow conditions in a dimension-confined semicircular channel designed with an ultra-low aspect ratio (1:9). Particularly, we achieved efficient fluid and particle manipulation in the designed microchannels by the systematic and precise generation and control of the multi-vortex. The results can enable an easy-to-fabricate/use fluid and particle manipulations with high and broad flow capacity and considerable flexibility in operation, as well as enhance the conceptual understanding of the helical vortex and Dean vortex manipulation. We expect that the ultra-low aspect ratio microchannel design will be easily integrated with other microfluidic components for a wide variety of applications in the biochemical, clinical, chemical engineering and environmental fields, such as for the highly efficient, low-cost and continuous isolation and detection of circulating tumor cells for clinical cancer diagnostics.

## Data Availability

Data sharing is not applicable to this article.

## Supplementary Materials

The following are available online at https://www.mdpi.com/article/10.3390/mi12070758/s1, Figure S1: Configuration of the four dimension-confined curved microfluidic channels (D1, D2, D3, D4). The D-channels are equipped with different amounts of ordered micro-bars, respectively. In D-channels, the narrow regions are all 450 μm wide, and the wide regions are all 900 μm wide. The channel heights are 100 μm in total. In order to identify the channel position, we define a coordinate system (x, y, z). The z axis points to the roof along the channel depth. The y axis always points to the channel wall perpendicular to the x axis, while the x axis refers to the main flow direction from the inlet to the outlet; Figure S2: Three operation modes (mode 3, mode 4, and mode 5) in the two dimension-confined curved microfluidic channels (D3++ and D4 +). D3++ channel is the design optimization of D3+. Based on the design of D3+ (all the inlets and outlets are 450 μm wide), we changed the dimensions of outer inlet and inner outlet by 90 μm in width and the inner inlet and outer outlet by 810 μm in width. Red dotted lines are used to analyze fluorescein distributions at the same positions. The analytical results are listed in Figure 4E and Figure 5A,B; Figure S3: Principle of contour diagram formation for the fluorescein trajectories in mode 5 under certain flow conditions (Re = 66.67 corresponding to flow rates 1 mL/min). For assessing the repeatability and stability of fluorescein trajectory, the contour diagram was chosen because it is able to present a combined fluorescein trajectory from lots of images for one manipulation experiment. Qualitative fluorescein trajectory can be depicted by contour diagram. In the beginning, a set of fluorescein images (5000–6000) were repeatedly captured at the same position from the same fluorescein operation test. The fluorescence images were then converted to grayscale images and each pixel’s data from the images was extracted by using software Image-Pro® Plus 6.0. Finally, the calculated total data from thousands of fluorescence images for the single test of fluorescein was normalized and converted to a single image (contour diagram) through software Origin 9. Scale bars, 300 μm; Video S1: Fluid mixing process of simulated concentration distribution in the four D-channels under a certain flow condition *Re* = 666.67 (Q = 10 mL/min); Video S2: Dynamic trajectories of fluorescein of the D3++ channel in operation mode 3 with rapidly changing Re. Time-lapse fluorescence images were continuously acquired when *Re* rapidly increased from 0 to 266.67 within 1.5 s and then decreased to the initial *Re.* The *Re* increasing from 0 to 266.67 was adjusted by directly setting the flow rate from 0 mL/min to 4 mL/min using a syringe pump; Video S3: Dynamic trajectories of fluorescein of the D4+ channel in operation mode 4 with rapidly changing Re. Time-lapse fluorescence images were continuously acquired when *Re* rapidly increased from 0 to 266.67 within 1.5 s and then decreased to the initial *Re*. The *Re* increasing from 0 to 266.67 was adjusted by directly setting the flow rate from 0 mL/min to 4 mL/min using a syringe pump; Video S4: Dynamic trajectories of fluorescein of the D4+ channel in operation mode 5 with rapidly changing *Re*. Time-lapse fluorescence images were continuously acquired when *Re* rapidly increased from 0 to 66.67 within 0.4 s. The Re increasing from 0 to 66.67 was adjusted by directly setting the flow rate from 0 mL/min to 1 mL/min using a syringe pump; Video S5: Stable trajectories of fluorescent particles (1% *w*/*v*) and fluorescein in the outlet regions of D3++ channel. Time lapse fluorescence images were continuously acquired when particles formed a single-band focusing at a high *Re* = 33.33 (0.5 mL/min); Video S6: Stable trajectories of fluorescent particles (1% *w*/*v*) and fluorescein in the outlet regions of D3++ channel. Time lapse fluorescence images were continuously acquired when particles formed a single-band focusing at a high *Re* = 133.33 (2 mL/min).
