# Supplementary material for: Multi-Vortex Regulation for Efficient Fluid and Particle Manipulation in Ultra-Low Aspect Ratio Curved Microchannels
Source: Micromachines (Basel). 2021 Jun 27;12(7):758. doi: 10.3390/mi12070758 (PMC8303296; doi:10.3390/mi12070758)
Supplement: Supplementary file 1 [file micromachines-12-00758-s001.zip › micromachines-1233460 supplementary for conversion.pdf]

micromachines

Supplementary Information: Multi-Vortex Regulation for Efficient Fluid and Particle Manipulation in Ultra-Low Aspect Ratio Curved Micro-Channels

Shaofei Shen *, Xin Wang and Yanbing Niu *

College of Life Science, Shanxi Agricultural University, Jinzhong 030801, China; wx15581740663@163.com
* Correspondence: shenshaofei@nwafu.edu.cn (S.S.); zhaol@nwafu.edu.cn (Y.N.);
Tel./Fax: +86-354-6287205 (S.S. &and Y.N.)

Abstract. This supplementary information provides all the additional information as mentioned in the text.

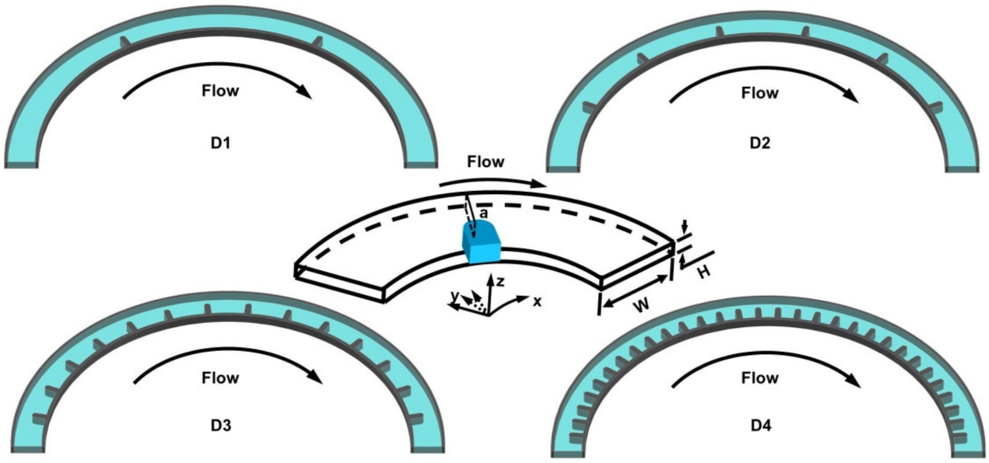

Figure S1. Configuration of the four dimension-confined curved microfluidic channels (D1, D2, D3, D4). The D-channels are equipped with different amounts of ordered micro-bars respectively. In D-channels, the narrow regions are all 450 µm wide, and the wide regions are all 900 µm wide. The channel heights are totally 100 µm. In order to identify the channel position, we define a coordinate system (x, y, z). The z-axis points to the roof along the channel depth. The y-axis always points to the channel wall perpendicular to the x-axis, while the x-axis refers to the main flow direction from the inlet to the outlet.

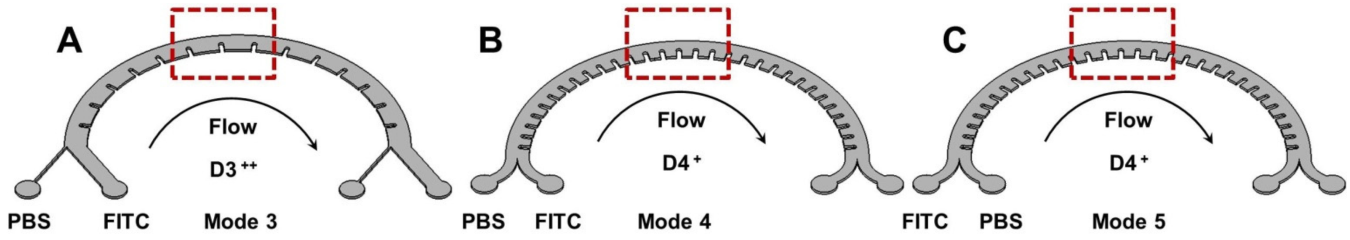

Figure S2. Three operation modes (mode 3, mode 4, and mode 5) in the two dimension-confined curved microfluidic channels (D3^{++} and D4 $^{+}$). D3^{++} channel is the design optimization of D3^{+}. Based on the design of D3^{+} (all the inlets and outlets are 450 μm wide), we changed the dimensions of outer inlet and inner outlet with 90 μm wide and inner inlet and outer outlet with 810 μm wide. Red dotted lines are used to analyze fluorescein distributions at the same positions. The analytical results are listed in Figures 4E and 5A,B.

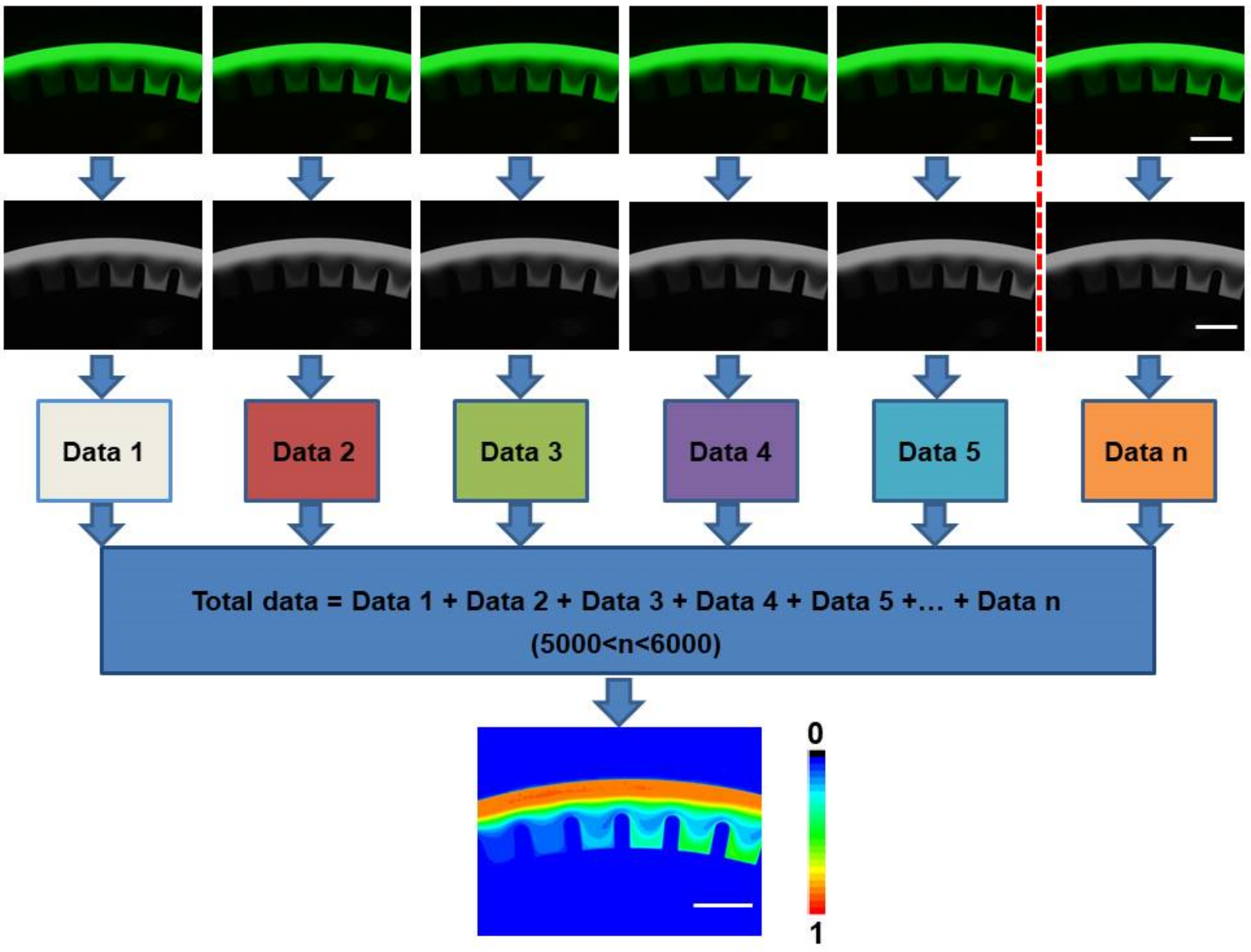

Figure S3. Principle of contour diagram formation for the fluorescein trajectories in mode 5 at a certain flow condition (*Re* = 66.67 corresponding to flow rates 1 mL/min). For assessing the repeatability and stability of fluorescein trajectory, contour diagram was chosen because that it is able to present a combined fluorescein

trajectory from lots of images for one manipulation experiment. Qualitative fluorescein trajectory can be depicted by contour diagram. In the beginning, a set of fluorescein images (5000–6000) repeatedly captured at the same position from the same fluorescein operation test. The fluorescence images were then converted to grayscale images and each pixel's data from images was extracted by using software Image-Pro® Plus 6.0. Finally, the calculated total data from thousands of fluorescence images for the single test of fluorescein was normalized and converted to a single image (contour diagram) by using software Origin 9. Scale bars, 300 μm.
